# Federated Learning in Healthcare Ethics: A Systematic Review of Privacy-Preserving and Equitable Medical AI

**DOI:** 10.3390/healthcare14030306

**Published:** 2026-01-26

**Authors:** Bilal Ahmad Mir, Syed Raza Abbas, Seung Won Lee

**Affiliations:** 1 Department of Electronics and Information Engineering, Jeonbuk National University, Jeonju 54896, Republic of Korea; bilalmir93@jbnu.ac.kr; 2 Department of Precision Medicine, Sungkyunkwan University School of Medicine, Suwon 16419, Republic of Korea; shahbaz5.12@skku.edu; 3 Department of Metabiohealth, Institute for Cross-Disciplinary Studies, Sungkyunkwan University, Suwon 16419, Republic of Korea; 4 Department of Artificial Intelligence, Sungkyunkwan University, Suwon 16419, Republic of Korea; 5 Personalized Cancer Immunotherapy Research Center, Sungkyunkwan University School of Medicine, Suwon 16419, Republic of Korea; 6 Department of Family Medicine, Kangbuk Samsung Hospital, Sungkyunkwan University School of Medicine, 29 Saemunan-ro, Jongno-gu, Seoul 03181, Republic of Korea

**Keywords:** federated learning, healthcare ethics, privacy preservation, algorithmic fairness, bias mitigation, data governance, medical AI, distributed machine learning, health equity

## Abstract

**Background/Objectives**: Federated learning (FL) offers a way for healthcare institutions to collaboratively train machine learning models without sharing sensitive patient data. This systematic review aims to comprehensively synthesize the ethical dimensions of FL in healthcare, integrating privacy preservation, algorithmic fairness, governance, and equitable access into a unified analytical framework. The application of FL in healthcare between January 2020 and December 2024 is examined, with a focus on ethical issues such as algorithmic fairness, privacy preservation, governance, and equitable access. **Methods**: Following PRISMA guidelines, six databases (PubMed, IEEE Xplore, Web of Science, Scopus, ACM Digital Library, and arXiv) were searched. The PROSPERO registration is CRD420251274110. Studies were selected if they described FL implementations in healthcare settings and explicitly discussed ethical considerations. Key data extracted included FL architectures, privacy-preserving mechanisms, such as differential privacy, secure multiparty computation, and encryption, as well as fairness metrics, governance models, and clinical application domains. **Results**: Out of 3047 records, 38 met the inclusion criteria. The most popular applications were found in medical imaging and electronic health records, especially in radiology and oncology. Through thematic analysis, four key ethical themes emerged: algorithmic fairness, which addresses differences between clients and attributes; privacy protection through formal guarantees and cryptographic techniques; governance models, which emphasize accountability, transparency, and stakeholder engagement; and equitable distribution of computing resources for institutions with limited resources. Considerable variation was observed in how fairness and privacy trade-offs were evaluated, and only a few studies reported real-world clinical deployment. **Conclusions**: FL has significant potential to promote ethical AI in healthcare, but advancement will require the development of common fairness standards, workable governance plans, and systems to guarantee fair benefit sharing. Future studies should develop standardized fairness metrics, implement multi-stakeholder governance frameworks, and prioritize real-world clinical validation beyond proof-of-concept implementations.

## 1. Introduction

The fast growth of digital health technologies has generated large amounts of medical data, creating both new opportunities for improved patient care and ethical challenges related to privacy, consent, and equitable access [1,2]. Machine learning (ML) and artificial intelligence (AI) algorithms have demonstrated strong potential in drug discovery, disease diagnosis, treatment recommendations, and costomize medicine [3,4]. The old centralized approach to ML, which gathers data from multiple sources into a single repository, faces significant obstacles in healthcare [5]. Strict privacy regulations, like GDPR in the European Union and HIPAA in the United States [6,7], institutional data sharing restrictions, and growing concerns about patient privacy [8,9,10] are key barriers to this approach.

Applying AI in healthcare raises fundamental ethical principles of accountability and equity, as algorithmic bias poses operational risks that may worsen existing disparities and undermine equitable care outcomes. ML models can unintentionally learn and reproduce systematic biases present in healthcare data through mechanisms such as unrepresentative training samples, historically biased clinical decisions encoded in labels, and differential data quality across demographic groups [11,12,13]. When biased models are deployed clinically, the principle of equitable care is threatened because these systems may produce differing diagnostic accuracy or treatment recommendations for different demographic groups [14,15]. Recent scholarship emphasizes that responsible medical AI implementation must extend beyond technical safeguards to include robust institutional governance, professional integrity, and the preservation of humanistic values [16].

By enabling collaborative model training across different institutions while keeping sensitive data localized, FL has emerged as a promising technique that partially addresses several of these concerns, though it does not eliminate all ethical challenges [17,18]. FL enables participating nodes to train models on their local datasets and share only model parameters or gradients with central aggregation server, which subsequently generates an updated global model that is distributed back among all participants [19,20]. Changing from data centralization to model propagation, this architecture radically changes the data governance structure, improving privacy protection while still allowing the creation of reliable, a widely applicable AI models trained on variety of datasets [21].

Although interest in federated learning for healthcare is rising, the literature is still split among technical, clinical, and ethical strands [22]. Many reviews focus on methods [18,23], privacy techniques [24,25,26], or particular clinical uses [27,28], but comprehensive, systematic analyses of the ethical issues are scarce. This review fills that gap by offering an integrated assessment of how federated learning confronts ethical challenges in healthcare AI, which are privacy preservation, fairness and bias mitigation, governance, transparency and explainability, and equitable access to AI technologies.

### 1.1. Comparison with Existing Reviews

Table 1 presents a detailed comparison between this systematic review and other recent reviews on FL in healthcare. Comparator reviews were selected based on (1) publication within the last three years, (2) focus on FL in healthcare settings, and (3) citation frequency in the field. This comparison highlights the unique contributions and focus areas of this work.

### 1.2. Contribution of This Review

This review condenses key ethical challenges and implementation-ready solutions for FL in healthcare, distinguishing actionable technical approaches from broader conceptual recommendations.

- We compare privacy methods (differential privacy, secure multi-party computation, homomorphic encryption, and hybrids) using three evaluation criteria: strength of privacy guarantees, computational overhead, and impact on model utility.
- Assess fairness methods across clients, demographic groups, and multi-objective settings, and critique commonly used metrics. We note that fairness metric definitions varied across studies, which we address as a limitation.
- Then, synthesize governance models, highlighting procedural, relational, and structural mechanisms for ethical FL deployment.
- Analyze strategies for equitable participation when institutions differ in compute, data quality, and expertise.
- HIghlight research gaps, absent standards, scarce clinical validation, limited patient based work, and tensions between fairness and privacy.

### 1.3. Research Objectives

This systematic review has four co-primary analytical objectives, treating privacy, fairness, governance, and equity as equally important ethical dimensions:

1. To synthesize privacy preservation techniques used in healthcare FL and assess their computational feasibility and trade-offs with model performance [9,24].
2. To analyze fairness and bias mitigation strategies across multiple levels (client-level, multi-dimensional, and attribute-level) and evaluate their impact on model equity and overall performance [11,30].
3. To examine governance frameworks and mechanisms that guide ethical implementation of FL in healthcare settings [29,31].
4. To assess strategies for equitable access and resource distribution in FL, particularly for institutions with limited computational capacity [19,32].

## 2. Methods

Systematic Reviews and Meta Analyses (PRISMA) 2020 standards [33] were followed in this systematic review to guarantee methodological, transparency, and reproducibility which PROSPERO registration is CRD420251274110. With clear focus on ethical issues like privacy, fairness, and governance, the review methodology was created to fully discover, assess, and synthesize evidence on FL applications in healthcare [17,29].

### 2.1. Search Strategy

A thorough literature search was conducted across several electronic databases [34]. PubMed/MEDLINE, IEEE Xplore Digital Library, Web of Science, Scopus, ACM Digital Library, SpringerLink and Science Direct, given the rapidly evolving nature of this field, arXiv was also searched to ensure comprehensive coverage; however, only preprints that had subsequently been published in peer-reviewed venues were included in the final analysis [6,7].

The search strategy used a combination of controlled vocabulary terms and keywords related to FL, healthcare applications, and ethical considerations [17]. The primary search string used Boolean operators to combine concepts: (“federated learning” OR “federated machine learning” OR “distributed learning” OR “collaborative learning” OR “decentralized learning”) AND (“healthcare” OR “medical” OR “clinical” OR “health” OR “biomedical” OR “hospital”) AND (“ethics” OR “fairness” OR “bias” OR “privacy” OR “equity” OR “governance” OR “transparency” OR “accountability”). The search was adapted for each database to account for differences in indexing systems and search syntax. All searches were conducted between September and October 2025. The complete search strategies for each database, including database-specific syntax and filters, are provided in Supplementary Table S1.

### 2.2. Eligibility Criteria

Studies were included in this review if they met the following criteria [33]:

Inclusion Criteria:

- Peer-reviewed journal articles or conference proceedings published between January 2020 and December 2024.
- Studies explicitly addressing FL methodologies or frameworks in healthcare or medical contexts.
- Research that incorporates or discusses ethical considerations not limited to privacy preservation, fairness, bias mitigation, governance, transparency, or equity.
- Original research articles, which are empirical studies, methodological developments, framework proposals, or systematic evaluations.
- Publications available in English language.
- Studies employing real-world healthcare data, synthetic medical datasets, or simulated federated healthcare scenarios.

Exclusion Criteria:

- Duplicate publications or multiple reports of the same study.
- Review articles, meta-analyses, opinion pieces, editorials, or commentaries (these were examined for reference mining but not included in primary analysis).
- Conference abstracts without full papers.
- Studies focusing solely on technical FL algorithms without healthcare application or ethical consideration.
- Research not available in English.
- Studies focusing exclusively on non-medical applications of FL.
- Book chapters, dissertations, and gray literature.
- Studies published before 2020 to ensure contemporary relevance.

### 2.3. Study Selection Process

The study selection process followed a systematic multi-stage approach [33]. Initial database searches yielded 3047 potentially relevant articles. Following removal of 612 duplicates using reference management software, 2435 unique articles remained for screening. Title and abstract screening was performed independently by two reviewers, with disagreements resolved through discussion or consultation with a third reviewer [17]. This process excluded 2167 articles that clearly did not meet inclusion criteria, leaving 302 articles for full-text review.

Full-text articles were assessed for eligibility against the predetermined inclusion and exclusion criteria [33]. During this stage, 230 articles were excluded for the following reasons: 87 had insufficient methodological detail, 74 had no clinical validation, 25 were not available in English, and 44 were published before 2020. This process resulted in 38 studies meeting all inclusion criteria for detailed data extraction and quality assessment.

### 2.4. Data Extraction

A standardized data extraction form was developed and piloted on five randomly selected included studies before full implementation [33]. Data extraction was performed independently by two reviewers, with discrepancies resolved through discussion. Extracted data elements included:

Study Characteristics: First author, publication year, country of origin, study design, healthcare domain, clinical specialty, sample size, and number of participating institutions [17].

Federated Learning Architecture: FL topology (centralized, decentralized, or hierarchical), distributed learning [35], aggregation algorithm, number of communication rounds, data partitioning approach (horizontal, vertical, or transfer learning), and handling of data heterogeneity [20,36].

Ethical Dimensions: Privacy-preservation techniques employed, fairness metrics and objectives, governance mechanisms, transparency and explainability methods, and stakeholder involvement strategies [24,29,30].

Privacy Techniques: Type of privacy-preservation method (differential privacy, homomorphic encryption, secure multi-party computation, etc.), privacy parameters, computational overhead, and privacy–utility trade-offs [9,24,37].

Fairness Approaches: Level of fairness addressed (client-level, attributelevel, multi-dimensional), fairness metrics used, bias mitigation techniques, and fairness-accuracy trade-offs [11,12].

Clinical Application: Medical domain (radiology, oncology, cardiology, etc.), task type (diagnosis, prognosis, treatment recommendation), data modalities (imaging, EHR, genomics, etc.), and reported clinical outcomes [38,39,40].

Performance Metrics: Model accuracy, area under the curve, F1-score, sensitivity, specificity, in fairness metrics like demographic parity, equalized odds, etc., privacy metrics like epsilon values for differential privacy, and computational efficiency measures [30,37].

### 2.5. Quality Assessment

Due to the heterogeneity of included study designs (methodological developments, empirical evaluations, framework proposals, and case studies), no single standardized quality assessment tool was applicable. Instead, studies were classified as high, moderate, or low quality based on author-defined criteria assessing (1) clarity of study objectives, (2) appropriateness of FL methodology, (3) depth of ethical considerations, (4) transparency in reporting results, and (5) presence of validation. Quality assessment was performed independently by two reviewers, with disagreements resolved through consensus discussion.

## 3. Results

### 3.1. Study Selection and Characteristics

**Figure 1:**
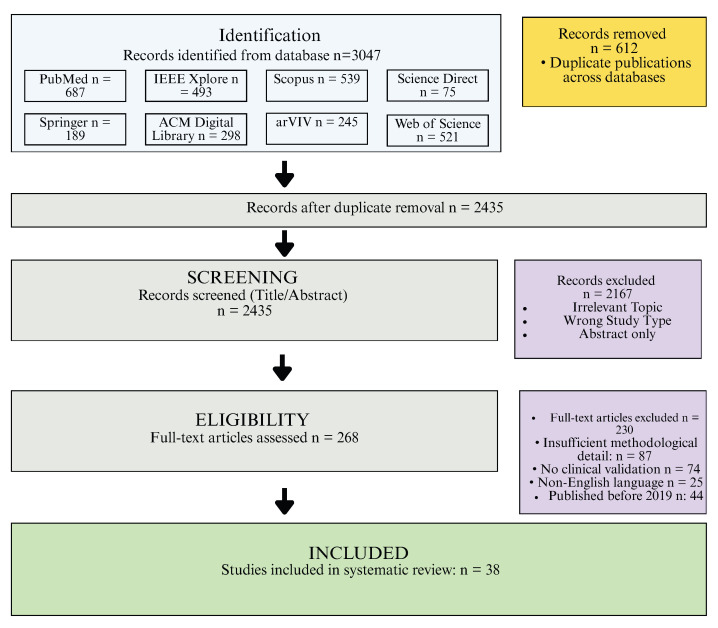
PRISMA 2020 flow diagram of study selection process.

The PRISMA flow diagram in Figure 1 shows the systematic study selection process. The initial database search identified 3047 potentially relevant articles. After removing 612 duplicates, 2435 unique articles remained for title and abstract screening, resulting in the exclusion of 2167 articles. Full text assessment of 268 articles led to the final inclusion of 38 studies Full text assessment of 302 articles led to the final inclusion of 38 studies that met all eligibility criteria and explicitly addressed ethical considerations in FL for healthcare.

Table 2 shows the key characteristics of the 38 included studies. The studies were published between 2020 and 2024, with a notable increase in publications after 2022, reflecting growing interest in ethical considerations of healthcare FL. Geographically, studies were classified by corresponding author affiliation, with the United States contributing 12 studies, making up 31.6%, China contributing 9, which is 23.7%. These two countries represent the largest contributors, followed by multi-national collaborations, which contributed 8, making up 21.1%, European countries, which contributed 6, making up 15.8%, and other regions contributing 3, which is 7.9%.

The included studies used various research designs, with methodological development studies used in 18 studies, representing 47.4%, followed by empirical evaluation, which were used in studies 12, which is 31.6%, framework proposals used in 6, 15.8%, and case studies used in 2, which is 5.3%. Medical imaging emerged as the predominant healthcare domain, and was used in 16 cases, which is 42.1%, followed by EHR, which was used in 11, 28.9%, with smaller representations from genomics and wearable device applications. Among clinical specialties, radiology was used in 9, 23.7%, and in 7, 18.4%. These were the most frequently addressed, reflecting the maturity of AI applications in these fields.

### 3.2. Privacy-Preservation Techniques in Healthcare FL

Privacy keeping appeared as central ethical consideration across all 38 included studies, though implementation approaches varied substantially. Table 3 summarizes the privacy preservation techniques used in included studies, their computational resources, and reported effectiveness.

Homomorphic encryption, which enables computations on encrypted data, was employed in 12 studies, representing 31.6%. While it offers strong theoretical privacy guarantees, it incurs significant computational overhead, with several studies reporting substantially increased training times compared to plaintext training.

A notable finding was the prevalence of hybrid privacy-preservation methods, observed in 18 studies, which make up 47.4%, which combine multiple techniques to balance privacy, computational efficiency, and model performance. These hybrid methods outperformed single technique implementations, providing robust privacy protection with minimal impact on model accuracy.

### 3.3. Fairness and Bias Mitigation in Healthcare FL

Fairness emerged as a critical ethical consideration, with 32 of 38 studies (84%) explicitly addressing algorithmic fairness or bias mitigation. Studies were considered to ’explicitly address fairness’ if they included fairness metrics, bias mitigation techniques, or substantive discussion of equity implications. The analysis revealed four distinct levels at which fairness was considered: client-level fairness, attribute-level fairness, multi-dimensional fairness (balancing multiple fairness objectives simultaneously), and intersectional fairness (addressing overlapping demographic vulnerabilities).

Table 4 summarizes key fairness levels explored in healthcare FL, along with corresponding metrics and mitigation strategies. The reported results indicate varying effectiveness across client, attribute, multi-dimensional, and intersectional fairness approaches.

Half of the studies (19 out of 38, 50.0%) tackled client-level fairness, grounded in the ethical principle that all participating institutions regardless of size or resources deserve equitably performing models for their patient populations, regardless of differences in their data distributions, quality, or volume. These methods included fair aggregation methods that weighted each institution’s contribution based on how well the model performed, rather than simply how much data they provided. The results showed substantial reductions in performance variations compared to standard federated averaging methods, with minimal sacrifice to overall model accuracy.

The most common focus area appeared in 22 studies are 57.9%, centering on reducing disparities in how models performed across different patient groups defined by characteristics like race, age, gender, and economic background. Researchers frequently measured fairness using demographic parity and equalized odds, with many studies tracking multiple fairness measures at once. The most popular technique for addressing these disparities was adversarial debiasing, which uses competing neural networks to prevent the model from making predictions based on sensitive attributes while maintaining its ability to accurately predict clinical outcomes. These methods reduced disparities across different demographic groups, though this sometimes came at the cost of modest reductions in overall accuracy.

Eleven studies explicitly recognized that healthcare machine learning systems must address fairness across multiple dimensions at once. The Federated Learning with Unified Fairness Objective framework marked notable progress by applying distributionally robust optimization to maintain consistent performance across all patient subgroups while sustaining acceptable overall accuracy. Evaluation results showed that these methods achieved more balanced improvements and retained overall model performance better than approaches targeting a single fairness objective.

One important limitation stood out, which was that only four studies considered intersectional fairness in terms of how multiple demographic characteristics combine to create distinct patterns of vulnerability. Since health disparities often hit hardest at intersection of multiple marginalized identities, this gap points to a important direction for future research.

### 3.4. Governance Frameworks for Ethical Healthcare FL

**Figure 2:**
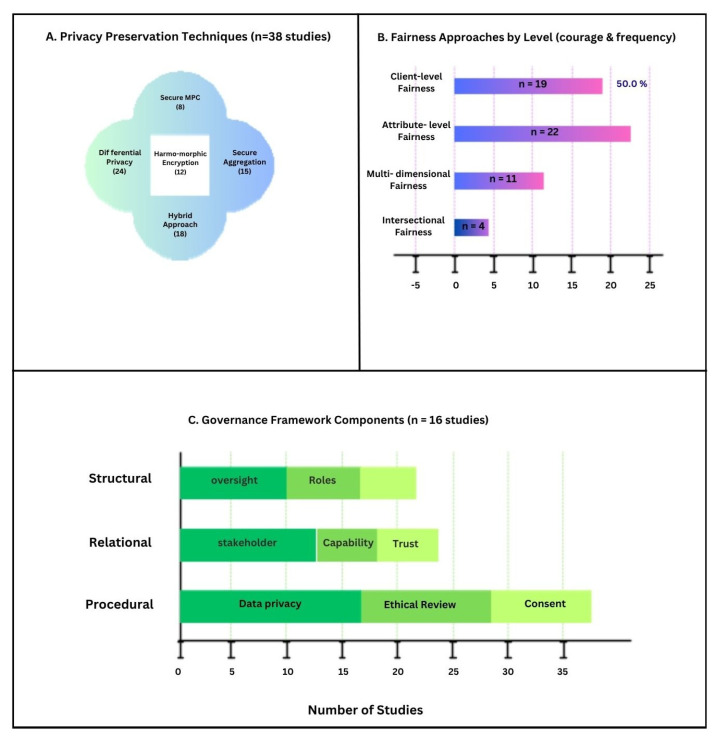
Distribution of ethical dimensions and study characteristics across all 38 included studies.

The analysis showed emerging but still underdeveloped governance frameworks for healthcare FL. Of the 38 included studies, 16 were be 42.1%, explicitly discussed governance mechanisms, with substantial heterogeneity in components and depth of governance considerations shown in Figure 2.

An overview of governance frameworks in healthcare FL is summarized in Table 5, outlining critical practices such as privacy protocols, ethical oversight, stakeholder participation, and audit-based accountability structures.

Procedural mechanisms, addressing how FL should be conducted, were most extensively developed. Data privacy protocols were discussed in 12 of the 16 governance-focused studies, typically incorporating principles of data minimization, purpose limitation, and secure communication. Nine governance-focused studies mentioned ethical review processes, though detailed implementation guidance was limited. Consent procedures for FL presented unique challenges, as traditional informed consent frameworks designed for centralized data collection may not adequately address the distributed nature and ongoing learning characteristics of FL systems.

Stakeholder engagement received moderate attention. Nine studies highlighted the need for interdisciplinary collaboration between ethicists, clinicians, data scientists, and patient representatives. Few offered concrete methods for stakeholder engagement across the FL lifecycle, such as documented consultation processes, patient advisory involvement, or feedback mechanisms. While capacity building and institutional support were identified as essential specially for resource-limited institutions detailed frameworks to address those capacity gaps were largely absent.

Defining organizational roles and oversight structures, were least developed. While seven studies (43.8% of governance-focused studies) mentioned the need for oversight bodies. Health consumer representation, emphasized as essential for patient-centered AI development, was explicitly addressed in only two studies (12.5% of governance-focused studies), representing a significant gap given the patient-facing nature of healthcare AI applications.

This lack of clear role definition creates significant accountability gaps, as it remains unclear how responsibility should be assigned when federated models cause patient harm whether to data providers, model developers, aggregation operators, or deploying institutions.

### 3.5. Comparative Analysis of FL Approaches

Table 6 presents a comparative analysis of different FL methods used in the included studies, evaluating their characteristics, advantages, challenges, and suitability for ethical healthcare applications.

Studies that were characterized by collaboration among a relatively small number of institutional participants emerged as the dominant and most ethically suitable approach for healthcare applications. This architecture aligns well with existing healthcare data governance structures, institutional privacy requirements, and regulatory frameworks. A total of 20 studies, which is 52.6%, used cross-silo FL, demonstrating practical feasibility in real-world healthcare settings.

Centralized FL with a trusted aggregation server was adopted in 25 studies (65.8%), providing an efficient and straightforward setup. This approach assumes the central server will not collude with adversaries or attempt to infer private information from received updates an assumption that may require institutional safeguards in healthcare settings. To mitigate these issues, several studies implemented secure aggregation protocols and encrypted communication, showing that centralized architectures can still deliver strong privacy protection when properly designed.

Decentralized designs that remove a central server were investigated in six studies, and are intended to increase trust and avoid single points of failure. However, their practical use in healthcare is limited by more complex coordination and consensus requirements and higher communication overhead barriers in contexts that require clear institutional oversight and accountability.

Hierarchical FL architectures, employing multiple levels of aggregation, were proposed in four studies, or 10.5%, as scalable solutions for large scale healthcare networks. These methods offered advantages for regional or national health systems.

Notably, none of the decentralized FL studies provided explicit governance mechanisms for handling disputes or coordinating model updates, representing a significant gap for ethical implementation.

### 3.6. Clinical Application Domains and Outcomes

Machine learning methods for genomics and epitranscriptomics, such as m5C-Seq and ORI-Explorer, further motivate privacy-preserving collaborative learning [41,42]. The included studies addressed diverse clinical applications, with varying depending on levels of maturity and real world implementation. Table 7 summarizes the primary clinical application domains, specific tasks, data modalities, and reported outcomes.

With 13 studies representing 35.1% of the most developed domain, medical imaging applications showed that FL models can achieve comparable performance to centralized models while preserving privacy, with most studies reporting only modest accuracy reductions. With discrepancy reductions of 30–50% across demographic categories, fairness interventions in imaging applications demonstrated promise. Nonetheless, there were still issues with addressing differences in label quality and guaranteeing representative training data from a variety of demographics.

Data heterogeneity, missing values, and complex temporal patterns were among the difficulties faced by EHR-based applications [43], which were analyzed in 11 studies (28.9%). On EHR data, privacy-preserving FL maintained reasonable accuracy levels comparable to centralized approaches, though performance varied depending on data heterogeneity and privacy mechanisms employed [44]. However, models often revealed significant performance disparities across racial, ethnic, and socioeconomic groups, raising ongoing concerns about fairness.

Since cancer care usually requires cooperation between specialist centers, each with distinct patient demographics and treatment methods, oncology applications (six studies; 15.8%) showed great promise for FL. While maintaining institutional autonomy and patient privacy, FL made it possible to establish multi-institutional models. Federated models for cancer diagnosis and therapy recommendation retained clinical utility comparable to centralized approaches while allowing validation across a variety of populations, according to several studies.

Six studies (15.8%) documented COVID-19 response applications, predominantly using retrospective data with limited real-time deployment, which showed the potential and limitations of FL in public health emergencies. Federated learning raised ethical questions about informed consent in emergency situations, equitable resource distribution, and guaranteeing model fairness when training data reflected disparate pandemic impacts across various communities, but it also made it possible for quick multi-institution collaboration to develop diagnostic and prognostic models without centralizing patient data.

### 3.7. Methodological Quality Assessment

The included studies’ methodological rigor and reporting transparency varied significantly, according to the quality evaluation. Twelve of the 38 studies, or 31.6% of the total, were deemed good quality due to their explicit study aims, suitable FL methodology, thorough ethical considerations, open reporting of privacy and fairness metrics, and thorough validation. Twenty studies, or roughly 52.6% of the total, were categorized as intermediate quality; they met most but not all quality standards, usually with limitations in validation techniques or the completeness of the ethical framework. Six studies, or around 15.8% of the total, were deemed to be of inferior quality and had serious flaws in their methodology, ethical considerations, or reporting openness.

Clear explanations of FL architectures, suitable aggregation algorithm selection, and methodical model performance evaluation were common methodological strengths. Inadequate discussion of generalizability to various healthcare contexts, limited long-term evaluation of fairness and privacy properties, limited real-world validation (with only five studies, or roughly 13.2%), and actual deployment in clinical settings were all commonly noted weaknesses.

## 4. Discussion

This systematic review provides the current state of FL applications that address ethical considerations including privacy, fairness, governance, and equity in healthcare settings, revealing both substantial progress and critical gaps. The synthesis of 38 studies demonstrates that FL offers technically viable and ethically potential approach for collaborative healthcare AI development, addressing fundamental challenges of privacy preservation, algorithmic fairness, and distributed governance. The translation from proof of concept research to real world clinical implementation remains limited, and significant ethical challenges require continued attention. To address the heterogeneity of included studies, we conducted stratified descriptive analyses by privacy technique in Table 3, fairness approach in Table 4, FL architecture in Table 6, and clinical application domain in Table 7, enabling identification of patterns across different methodological categories.

### 4.1. Privacy-Preservation: Balancing Theoretical Guarantees with Practical Utility

Healthcare AI is starting to incorporate privacy-preserving strategies like safe aggregation and differential privacy, signaling a change from treating privacy as an afterthought to including it into model design [45,46]. Moderate epsilon levels, such as 1.0–5.0, typically maintain adequate accuracy, although striking a balance between privacy and model utility continues to be a major difficulty, despite the fact that privacy requirements may vary across clinical contexts and patient populations, the included studies rarely incorporated patient perspectives into privacy budget decisions [47,48].

Although homomorphic encryption provides robust privacy protection, its 10×–100× computational cost makes it unfeasible for large-scale healthcare models. Although they increase implementation complexity, hybrid techniques that include safe aggregation, trusted execution environments, or differential privacy show promise by increasing privacy with little performance cost. Inadequate assessment of privacy concerns unique to FL, such as gradient leakage and model inversion attacks [49,50,51], is a significant unresolved issue.

Privacy budget parameters (e.g., epsilon values for differential privacy) were typically selected by researchers based on prior literature or empirical utility-privacy trade-off experiments, with limited justification for clinical appropriateness.

### 4.2. Algorithmic Fairness: From Single Metrics to Multi-Dimensional Equity

With 84% of studies specifically addressing fairness, the importance of fairness in healthcare AI is becoming more widely acknowledged. Significant advancements, institutional discrepancies, and attribute levels are all simultaneously marked by the transition from single metric to multi dimensional fairness frameworks. Fair aggregation techniques reduce variation by 40–60% without significantly compromising performance, and client-level fairness guarantees consistent model performance across healthcare facilities serving heterogeneous populations. Adversarial debiasing and other attribute-level techniques reduced demographic inequalities by 30–70%, although they frequently resulted in an accuracy drop of 2–8%. Focusing solely on age, gender, or race may overlook other sources of disparity such as geographic, socioeconomic, or institutional factors that influence healthcare outcomes [52,53].

By jointly optimizing several equity goals using distributionally balancing fairness and utility more successfully than sequential methods, multidimensional fairness frameworks enhanced this research. Practical use is hampered by their complexity [54,55,56]. Strict fairness restrictions reduced performance for majority groups, according to some research, sparking moral discussions about striking a balance between equity and equality. These difficulties show that ethical reasoning and community-based principles must be in line with technical solutions in order for healthcare AI to be fair.

### 4.3. Governance: From Technical Solutions to Institutional Frameworks

There is a glaring disconnect between institutional preparedness for ethical implementation and technical advancements in FL, as only 42% of research specifically addressed governance. Although techniques for privacy and justice have improved, there are still few frameworks for monitoring, consent, and accountability. Liability is uncertain when models cause harm since traditional informed consent and review procedures frequently ignore FL’s shared duties and continuous model updates [57,58]. Smaller or less-resourced institutions are at a disadvantage since relational issues like stakeholder involvement and capability building have not received as much attention in governance discussions as procedural procedures like data privacy.

Only two studies (5.3%) explicitly included patient representation, although Table 3 indicates that 25% included some form of patient input, and structural governance systems that define responsibilities and decision-making procedures are still in their infancy. Although they frequently function without standardized frameworks, which limits interoperability, emerging healthcare FL consortia offer examples of collaborative governance [59]. Federation opacity the inherent difficulty in auditing model behavior and tracing data contributions across distributed nodes without centralizing sensitive information represents a significant ethical challenge for transparency and accountability.

### 4.4. Clinical Translation: Bridging the Gap Between Research and Practice

Only 13% of studies reported real world clinical deployment, highlighting a major translational gap: proof-of-concept and simulated federations miss organizational, regulatory, legal, and human-factor barriers that arise in practice [60,61]. Medical imaging has demonstrated strong performance in advanced ultrasound imaging tasks [62,63] and appears closest to readiness federated imaging models often reach 85–95% of centralized performance likely thanks to standardized data and established multi-center collaborations, but scanner heterogeneity, population representativeness, and cross-site validation remain unresolved.

EHR-based FL faces larger hurdles due to heterogeneous systems, variable documentation and coding, and entrenched biases that make fairness hard to achieve [64]. Specialties with strong multicenter networks, like oncology and cardiology, show promise but contend with intellectual property, competition between systems, and transparency concerns. Federated efforts during COVID-19 demonstrated speed and privacy advantages but also exposed risks from compressed governance and fairness shortcuts when urgency overrides deliberative oversight.

### 4.5. Limitations

This systematic review has several limitations that should be considered when interpreting findings that should be considered when interpreting findings. Geographic bias may have been introduced by the restriction to English-language publications, which may have left out pertinent studies written in other languages. Quantitative meta-analysis was not possible due to the diversity in FL techniques and ethical frameworks among studies, which limited our capacity to draw firm comparative conclusions regarding the efficacy of various approaches.

The emphasis on ethical issues inevitably draws attention to normative issues that technical research may subtly address using various terminologies, which could have an impact on study inclusion choices. Conclusions from earlier studies may not accurately reflect contemporary ethical requirements or best practices due to the rapid changes in privacy rules, fairness standards, and governance expectations.

### 4.6. Implications for Practice and Policy

**Figure 3:**
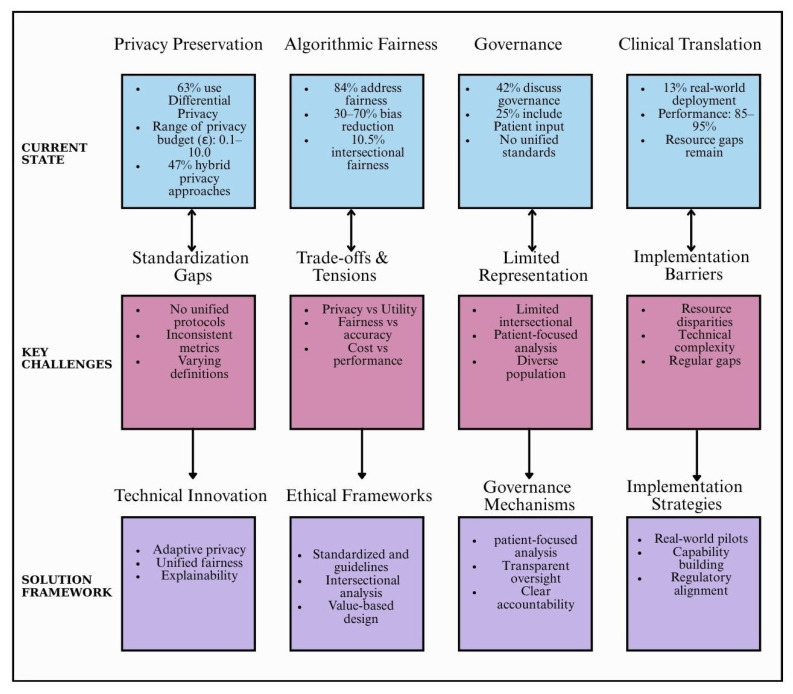
Key challenges and solution framework for ethical healthcare FL.

Treat privacy as mandatory for embed privacy-preserving techniques and set context-specific privacy budgets aligned with patient preferences and regulations [65]. Figure 3 summarizes the key challenges identified in this review and presents a solution framework for ethical healthcare FL implementation.

Make a fairness routine for evaluating models across institutions and clinical subpopulations; choose metrics by ethical and clinical priorities and manage trade-offs explicitly.

Define governance up front which sets clear roles, decision processes, accountability, and include patient and stakeholder representation; build an institutional capacity for equitable participation.

Update regulation sensibly for clarify data permissions, liability, and approval paths for FL while avoiding unnecessary barriers; encourage regulatory harmonization for cross-border collaborations.

Run real-world pilots for prioritize evaluation of performance, privacy, fairness, governance, and user experience, and share results to speed up broader, safer adoption.

### 4.7. Future Directions

- Unified ethical frameworks combining technical, governance and consent standards.
- Privacy methods tuned for clinical data (efficient HE/SMP, hybrid verification).
- Intersectional fairness that includes social determinants of health.
- Patient-centered governance: clear explanations, consent, and ongoing engagement.
- Resource-efficient FL approaches to enable equitable participation by low-resource and community institutions, measured by computational requirements, technical expertise needed, and infrastructure demands.
- Real-world validation via pilot/pragmatic trials and longitudinal monitoring.
- Federated explainability and transparency standards without centralizing data.
- Standardized benchmarks including reference datasets, evaluation protocols for privacy–utility trade-offs, fairness assessment procedures, and governance compliance checklists.

## 5. Conclusions

This systematic review, conducted according to PRISMA 2020 and drawn from 3047 initially identified records, found that FL is both technically feasible and ethically promising for collaborative healthcare AI, but important translational gaps remain. Privacy methods such as differential privacy and secure aggregation are becoming more established; hybrid approaches that combine techniques can provide strong protection with only modest losses in utility. Multi-dimensional fairness strategies show promise for reducing disparities, though achieving fairness gains often requires accepting accuracy trade-offs. Real-world clinical deployment remains limited, and governance frameworks are underdeveloped, with insufficient attention to oversight and accountability mechanisms, and patient and community engagement is largely absent. There are no widely adopted ethical standards for healthcare FL, which leads to inconsistent implementation and evaluation. It will be necessary to include privacy and fairness mechanisms into explicit governance structures that specify responsibilities, accountability, and stakeholder participation in order to move FL from research to reliable practice. The central insight from this review is that technical advances in privacy and fairness must be matched by equally rigorous governance frameworks and stakeholder engagement to realize FL’s ethical potential in healthcare. Real-world pilots that assess technological performance in addition to privacy attributes, fairness results, governance efficacy, and user experience should be prioritized. To facilitate cross-institutional cooperation without creating needless obstacles, standardized ethical frameworks and regulatory guidelines are required. In order to reach a consensus on acceptable privacy–utility trade-offs, fairness goals, and equitable benefit sharing, physicians, data scientists, ethicists, legislators, and patient representatives must collaborate on these technical and normative decisions. As healthcare systems increasingly adopt AI technologies, FL stands at a critical juncture: with sustained interdisciplinary collaboration, patient-centered governance, and commitment to rigorous real-world validation, it can fulfill its promise of enabling equitable, privacy-preserving medical AI that serves all patient populations.

## Figures and Tables

**Table 1:** Contribution comparison: this review vs. existing reviews.

Review	Primary Focus	Ethical Emphasis	Studies Included	Key Limitations
[17]	Clinical applications and technical architecture	Limited (privacy only)	612	Minimal fairness/governance focus; broad scope.
[8]	IoT integration, privacy, and security	Moderate (privacy and security)	Not specified	Limited fairness analysis; IoT-specific.
[19]	Challenges and recommendations	Moderate (privacy concerns)	107	Limited governance frameworks; technical focus.
[18]	Methodological advances	Limited (privacy techniques)	89	Minimal ethical framework; methods-focused.
[24]	Privacy preservation techniques	High (privacy only)	Review article	No fairness/governance; privacy-specific.
[29]	Governance mechanisms	High (governance only)	39	Limited privacy/fairness integration.
This Review	Comprehensive ethical healthcare FL	Very High (all dimensions)	38	Integrates privacy, fairness, governance, and equity; PRISMA-compliant; multi-dimensional analysis.

Note: Ethical emphasis categories (Limited, Moderate, High, Very High) were author-defined based on the extent to which each review addressed privacy, fairness, governance, and equity dimensions. Ethical Emphasis categories were author-defined: ’Limited’ = addresses one ethical dimension minimally; ’Moderate’ = addresses two dimensions; ’High’ = addresses three dimensions comprehensively; ’Very High’ = comprehensively addresses all four dimensions (privacy, fairness, governance, and equity) with integrated analysis.

**Table 2:** Characteristics of included studies (n = 38).

Characteristic	Number of Studies	Percentage (%)
Publication Year		
2020	3	7.9
2021	4	10.5
2022	6	15.8
2023	14	36.8
2024	11	28.9
Geographic Region		
United States	12	31.6
China	9	23.7
Multi-national	8	21.1
Europe	6	15.8
Other	3	7.9
Study Type		
Methodological development	18	47.4
Empirical evaluation	12	31.6
Framework proposal	6	15.8
Case study	2	5.3
Healthcare Domain		
Medical imaging	16	42.1
Electronic health records	11	28.9
Genomics	4	10.5
Wearable devices/IoT	4	10.5
Multi-domain	3	7.9
Clinical Specialty		
Radiology	9	23.7
Oncology	7	18.4
Cardiology	5	13.2
Internal medicine	4	10.5
Neurology	3	7.9
Multiple specialties	10	26.3

**Table 3:** Privacy-preservation techniques in healthcare federated learning.

Technique	Studies (n)	Privacy Guarantee	Computational Overhead	Utility Trade-Off	Healthcare Applicability
Differential Privacy (DP)	24	DP	Low to Moderate	Moderate	High
Homomorphic Encryption	12	Information-theoretic	High	Low	Moderate
Secure Multi-party Computation	8	Information-theoretic	Very High	Low to Moderate	Low
Secure Aggregation	15	Computational	Low	Very Low	High
Trusted Execution Environment	4	Hardware-based	Moderate	Low	Moderate
Blockchain Integration	6	Cryptographic	High	Moderate	Moderate
Synthetic Data Generation	5	Semantic	Low	Moderate to High	High
Hybrid Approaches	18	Combined	Variable	Variable	High

Note: Overhead, utility trade-off, and applicability are summarized qualitatively based on how each study reported runtime/communication cost, performance changes versus non-private baselines, and feasibility in real healthcare settings.

**Table 4:** Fairness approaches and metrics in healthcare federated learning.

Fairness Level	Studies (n)	Primary Metrics	Mitigation Strategies	Reported Effectiveness
Client-Level Fairness	19	Performance variance, worst-case accuracy, Gini coefficient	Fair aggregation, client weighting, resource allocation	High (variance reduction 40–60%)
Attribute-Level Fairness	22	Demographic parity, equalized odds, equal opportunity	Adversarial debiasing, reweighting, fair representation learning	Moderate to High (disparity reduction 30–70%)
Multi-Dimensional Fairness	11	Harmonic mean of fairness metrics, Pareto frontier analysis	Unified fairness objectives, constrained optimization	Moderate (balanced improvement 25–45%)
Intersectional Fairness	4	Subgroup-specific metrics	Hierarchical fairness constraints	Limited evidence

**Table 5:** Governance mechanisms in healthcare federated learning.

Governance Component	Studies (n)	Key Elements
Procedural Mechanisms		
Data Privacy Protocols	12	Data minimization, purpose limitation, access controls, encryption standards
Ethical Review	9	IRB approval, ethics committee oversight, ethical impact assessment
Consent Procedures	5	Informed consent frameworks, opt-in/opt-out mechanisms, consent for secondary use
Formal Agreements	6	Data sharing agreements, participation contracts, liability frameworks
Relational Mechanisms		
Stakeholder Involvement	9	Clinician engagement, patient representatives, ethics practitioners
Capability Building	7	Training programs, technical support, knowledge sharing
Institutional Support	6	Resource allocation, infrastructure investment, organizational commitment
Trust Building	5	Transparency initiatives, communication protocols, dispute resolution
Structural Mechanisms		
Oversight Bodies	7	Federated learning consortia, governance boards, regulatory compliance structures
Role Definition	6	Data custodian roles, model developer responsibilities, end-user accountability
Health Consumer Representation	2	Patient advisory boards, community engagement, feedback mechanisms
Audit Mechanisms	6	Regular audits, performance monitoring, fairness assessments

Note: Study counts reflect the frequency with which each governance component was discussed or proposed in the included studies, not necessarily evidence of real-world implementation.

**Table 6:** Comparative analysis of federated learning approaches in healthcare.

FL Approach	Studies (n)	Key Advantages	Ethical Challenges	Healthcare Suitability
Centralized FL	25	Simple coordination, efficient aggregation, established protocols	Single point of failure, server trust requirements, potential privacy risks	High for institutional networks
Decentralized FL (P2P)	6	No central authority, enhanced resilience, distributed trust	Complex coordination, higher communication costs, consensus challenges	Moderate for multi-institutional
Hierarchical FL	4	Scalability, regional aggregation, flexible architecture	Complex governance, multi-level fairness considerations	High for large-scale deployments
Cross-Silo FL	20	Institution-level privacy, manageable participants, stable infrastructure	Data heterogeneity, fairness across institutions	Very High for healthcare
Cross-Device FL	4	Large-scale deployment, individual privacy	Device heterogeneity, limited healthcare validation	Moderate for wearables/mobile health
Vertical FL	3	Feature partitioning, complementary data	Complex privacy preservation, limited healthcare applications	Low currently
Federated Transfer Learning	7	Knowledge sharing across domains, limited data requirements	Domain shift challenges, fairness across source/target	Moderate for rare diseases

**Table 7:** Clinical application domains in healthcare federated learning studies.

Domain	Studies (n)	Primary Tasks	Data Modalities	Key Findings
Medical Imaging	13	Lesion detection, tumor segmentation, disease classification	X-ray, CT, MRI, pathology images	FL models achieved 85–95% of centralized performance; fairness interventions reduced disparities by 30–50%.
Electronic Health Records	11	Risk prediction, treatment recommendation, readmission forecasting	Structured EHR, clinical notes	Privacy-preserving FL maintained 80–92% accuracy; significant fairness challenges across demographic groups.
Oncology	6	Cancer detection, treatment response prediction, survival analysis	Multi-modal: imaging + genomics + EHR	Federated models demonstrated clinical utility; privacy techniques showed minimal impact on performance.
Cardiology	5	Cardiovascular risk assessment, ECG analysis, heart disease prediction	ECG signals, cardiac imaging, EHR	FL enabled multi-center validation; fairness-aware training reduced gender disparities.
Genomics	4	Genetic risk scoring, biomarker discovery, pharmacogenomics	Genomic sequences, SNP data	Secure genomic FL feasible but computationally intensive; privacy critical given genetic data sensitivity.
Wearables/IoT	4	Health monitoring, early warning systems, chronic disease management	Sensor data, activity logs	Demonstrated feasibility for patient-centric FL; privacy preservation essential.
COVID-19 Response	6	Diagnosis, prognosis, resource allocation	Chest imaging, EHR, mobility data	Rapid deployment demonstrated FL agility; ethical challenges in crisis contexts identified.

Note: Study counts reflect the specific clinical application tasks included in this table and may differ from counts reported using broader domain groupings elsewhere in the manuscript.

## Data Availability

No new data were created or analyzed in this study.

## Supplementary Materials

The following supporting information can be downloaded at: https://www.mdpi.com/article/10.3390/healthcare14030306/s1, Table S1: Database search strategy and retrieval counts.

## Abbreviations

The following abbreviations are used in this manuscript:

FL	Federated Learning
AI	Artificial Intelligence
ML	Machine Learning
DP	Differential Privacy
HE	Homomorphic Encryption
SMPC	Secure Multi-Party Computation
EHR	Electronic Health Records
PRISMA	Preferred Reporting Items for Systematic Reviews and Meta-Analyses
PROSPERO	International Prospective Register of Systematic Reviews
JBI	Joanna Briggs Institute
IoT	Internet of Things
P2P	Peer-to-Peer
