# Supplementary material for: Federated Learning in Healthcare Ethics: A Systematic Review of Privacy-Preserving and Equitable Medical AI
Source: Healthcare (Basel). 2026 Jan 26;14(3):306. doi: 10.3390/healthcare14030306 (PMC12896918; doi:10.3390/healthcare14030306)
Supplement: Supplementary file 1 [file healthcare-14-00306-s001.zip › healthcare-4079472-supplementary.pdf]

Supplementary Material

Search Strategy Details

Table S1. Database search strategy and retrieval counts

[flushleft]

Note: "n" indicates the number of records retrieved at the time of search.

Table S1: Database search strings, filters applied, search dates, and records retrieved.

**Database (n)**<br>**Search date**	**Search string**	**Filters applied**
PubMed (n=687)<br>September 15, 2024	(`"federated learning"` OR `"federated machine learning"` OR `"distributed learning"`) AND (`"healthcare"` OR `"medical"` OR `"clinical"`) AND (`"ethics"` OR `"fairness"` OR `"bias"` OR `"privacy"` OR `"governance"`)	English language; 2020–2024
IEEE Xplore (n=493)<br>September 16, 2024	(`"federated learning"` OR `"distributed learning"`) AND (`"healthcare"` OR `"medical"`) AND (`"ethics"` OR `"fairness"` OR `"privacy"`)	Conference papers and journals; 2020–2024
Scopus (n=539)<br>September 17, 2024	Same Boolean string adapted to Scopus syntax	English; Article/Review; 2020–2024
Web of Science (n=521)	Same Boolean string	English; Article; 2020–2024

Continued on next page

**Database (n) Search date**	**Search string**	**Filters applied**
September 18, 2024		
ACM Digital Library (n=298) September 19, 2024	Same Boolean string	2020–2024
arXiv (n=245) September 20, 2024	Same Boolean string	cs.LG and cs.AI categories; 2020–2024
Springer (n=189) September 21, 2024	Same Boolean string	English; 2020–2024
ScienceDirect (n=75) September 22, 2024	Same Boolean string	Research articles; 2020–2024
